# Supplementary material for: Qualitative Study of Maternity Healthcare Vulnerability Based on Women’s Experiences in Different Sociocultural Context
Source: Nurs Rep. 2025 Mar 18;15(3):105. doi: 10.3390/nursrep15030105 (PMC11945914; doi:10.3390/nursrep15030105)
Supplement: Supplementary file 1 [file nursrep-15-00105-s001.zip › nursrep-3468836-supplementary.pdf]

Table S1. Semi-structured in-depth interview guide that includes the questions asked to women.

Thank you for taking your time to meet me today. My name is Claudia, and I am a PhD-student from the Universidad Autónoma de Madrid (Spain). For my PhD project, I conduct research on maternal health in Spain in collaboration with Colombia. For this research, I am interested in your experiences and perceptions of maternity rights in terms of maternal health services. This interview consists of two parts. With your permission I would like to audio-recorder the interview because I do not want to miss any of your comments. [*Turn audio recording device on*] All responses will be kept confidential. This means that your interview responses will only be shared with research team members. Any personal information in the final report will not identify you as the respondent. Furthermore, the recording will also be deleted when reports have been written. We hope that this research with your help will benefit other women. However, for yourself personally there will be no benefits but also no risk of taking part in the research. I emphasise that you do not have to talk about anything you do not want to. You can stop participation any time and without any explanation. Do you have any questions about this explanation? Are you willing to participate? If you have any questions during our conversations, please do not hesitate to ask.

Start Interview

The perception of OB/GYN vulnerability: concepts and experiences.

- Kindly explain what your experience was in the care process during your last pregnancy, childbirth, and late postpartum? Was it satisfactory?

Examining questions:

  - *[If note dissatisfaction in the response]* What would you change about your experience to make you feel more satisfied?
  - *[If note satisfaction in the response]* What would you keep from your experience to have a greater sense of satisfaction?

- When you knew you were pregnant, what were your expectations regarding the healthcare you would receive?
- Were the expectations you mentioned met? Please, kindly explain why.
- How reasonable do you think those expectations were to be met?

Examining questions:

  - *[If expectations were unreasonable]* Would there be anything to make these expectations real?

- What do you know about the violence or abuse that women can experience during their gynecological-obstetric care?

Examining questions:

- *[If woman knows about the concept]* Did you experience any situation of violence or abuse during your healthcare? Or heard of someone close to you experiencing it? Could you kindly describe the situations you experienced or know?

Factors that influence the perception of OB/GYN vulnerability.

Next, I am interested in knowing the conditions of any kind, yours, your environment, your family, that affect your experience with motherhood.

- What do you think influences the situations of violence or abuse?

- What conditions do you think benefited to have a positive experience during healthcare?
- What conditions do you think did **not** benefit to have a positive experience during healthcare?

The perception of maternity rights.

Like I explained before, I am interested in the maternity rights in the healthcare setting.

- What do you think are the rights that women have during pregnancy, childbirth and postpartum care?
- Do you think a woman can decide during her care? And what do you think are exclusive decisions of health providers?

Examining questions:

- *Is there any exception to women's rights being fulfilled in clinical practice, which is normatively justifiable?*
- *What are the rights that cannot be unfulfilled?*

The perception of motherhood

The following questions refer to your overall experience in motherhood.

- What does "*be a mother*" mean to you? Or what is the most important thing about "*be a mother*"?
- Regardless of whether the pregnancy was planned or not, why did you decide to be a mother?
- How has motherhood changed you since you knew about pregnancy today?
- Would you change anything about who you are today? Please, kindly explain why.

End interview

Do you have any additional thoughts you would like to share? Or do you have any comments or questions concerning the interview?

Thank you for your time.

[*Turn audio recording device off*]

Table S2. The original Spanish-transcript sections from women's interviews (C=Colombian, S=Spanish).

Transcript I.
"He escuchado, en las mujeres que tienen muchos hijos, que los médicos empiezan a generar ese rechazo de que quedan embarazadas a cada rato, y empiezan a hacer comentarios que menosprecian esa condición". (C3)
"Cuando tienen que hacer instrumentación en el parto de la manera en la que lo hacen". (S3)

Transcript II.
"Vi muchos casos en los que eran las 3 o 4 de la tarde, todo el día con hambre, no las habían pasado a una sala de cirugía porque no había un orden". (C2)
"El doctor que me atendió esa vez no sé qué tenía en la mente. Me hizo una ecografía y me dijo: no, el bebé se está gestando en la cicatriz de la cesárea, no puede nacer; en esta semana yo creo que se va a venir solo o sino véngase tal día y yo le practico el aborto porque no puede nacer. Otro doctor me vio, me hizo una ecografía y me dijo que no, que había un desprendimiento de 4 milímetros, pero que con hormonas y cuidados iba a estar bien. Y así fue." (C1)
"Yo pensaba: pues me provocan el parto, están ahí encima y está todo muy controlado. Pero en realidad no es así. A mí me bajaba la tensión y nadie se enteraba". (S1)

Transcript III.
"Te hablan: es su culpa, usted se lo buscó. Literal, a mí una enfermera me dijo: pues para qué abrió las piernas". "Que no te escuchen, que no te pongan atención simplemente porque: él es el médico. Una escucha su cuerpo y entiende cosas, una sabe que algo está cambiando y que algo de no va bien". (C2)
"Han dicho: es que estás más gorda que una cerda, así de gorda no puedes estar, como una foca. ¡Cosas feas!". (S1)
"El derecho de ir a que la atiendan en cualquier lado, porque a veces vas y necesitas sacar la tarjeta de salud, pero no puedes si no tienes el pasaporte vigente, o sea, mucha documentación". (S2)

Transcript IV.
"Esos tactos que le hacen a una a cada rato… la verdad es muy traumático… No sé si eso medicamente sea necesario o exista otra manera… sentía que me metía la mano, el puño y el codo". (C3)
"Como están mucho por el parto natural, que te aguantan hasta casi morir para que nazcan naturales. No sé, que prevalezca lo de la madre". (S1)
"Como no sabía, yo empujaba y por la epidural no hacía la fuerza suficiente.... Los médicos se subieron encima y con el brazo me apretaron en la barriga para que saliera y eso lo recuerdo super fatal… si no es que el bebé se va a morir que nadie me haga esto, porque lo pasé mal y es una sensación incómoda, estás en un momento bonito y estás ahí agonizando, porque te duele que te aprieten en el estómago, no te duele el momento de parir". (S3)

Transcript V.
"Una enfermera dijo: usted abandonó a su hija acá. Económicamente ella no sabía cómo estaba, emocionalmente ella no sabía cómo estaba. Por mi hubiera pasado las 24 horas del día allá, pero

en mi casa tenía otra bebé recién nacida, no tenía quién la cuidara, y la que estaba en la clínica sabía que estaba rodeada de profesionales. Yo iba dos veces al día. La verdad me sentí super mal, no abandoné a mi hija, simplemente no podía hacerlo". (C2)
"Son muy de que tienes tú el hijo y tú tienes que saber, pero no se nace sabiendo. Tener un de ponerse en el lugar de la mujer que recién ha parido o que no sabe a veces ni cargarlo". (S2)
"Estoy súper preocupada por mi bebé porque en el hipotético caso de que tenga Síndrome de Down, ¿qué hago?, ¿sigo adelante o no?, Y el otro *(refiriéndose al profesional sanitario)* solo piensa en los 600 euros de la prueba". (S3)

Transcript VI.
"Que se sobrepasan con las mujeres, digamos en tocarlas físicamente o en opinar sobre su apariencia física". (C1)
"Simplemente te dan una palmadita y prepárate para no dormir. Una dice: ¿cómo así para no dormir?". No te imaginas la magnitud de lo que se viene. Que digan: no vas a dormir por esto, organiza tus tiempos, prepárate así, haz una rutina así…". "Me decía: un embarazo normal es solo un bebé, aquí ya empezamos mal *(refiriéndose a embarazo gemelar)*." (C2)
"La primera vez me salió positivo para toxoplasmosis, no me supo explicar bien, y la verdad yo salí asustada, llorando". (C3)
"Lo malo es que no tienes una persona de confianza que vayas siempre a la misma, se guían por lo que ha dejado escrito la anterior". "Yo pedí: por favor, es que me encuentro fatal, si ya he dilatado totalmente y la niña no ha bajado, habrá alguna solución. Como si no me hubieran oído". (S1)

Transcript VII.
"En niñas o en mujeres muy jóvenes embarazadas o que viven en un pueblo, que conocen muy poco". (C1)
"Que la maternidad era lo más hermoso del mundo y no, la depresión postparto existe. La maternidad la han romantizado muchísimo". "Es muy duro, es muy *heavy*, son tres meses en que no duermes, literal no duermes nada, y tienes que responder por dos bebés, cuando muchas veces uno ni sabe responder por uno mismo". (C2)
"Uno piensa que todo es automático cuando tiene el primer niño y no, hay cosas que definitivamente son bastante complejas" (C3)
"Estás con las puertas abiertas, ¿qué estás oyendo?: paritorio número no sé, corriendo a quirófano… aún te genera más ansiedad porque encima no te encuentras bien, tienes miedo porque no sabes lo que va a pasar realmente al final y encima estás oyendo cosas externas que no sabes si te van a pasar a ti también". "Cuando estás embarazada cada uno te dice una cosa, realmente no sabes a dónde vas". (S1)
"Uno quizás deja de lado lo suyo, quizás lo profesional en mi caso, y por velar porque ellos no se queden solos o no estén todo el día en la guardería o con otras personas, es un poco de sacrificio". (S2)
"Es que tienes las emociones tan a flor de piel, es que eso es incontrolable". (S3)

Transcript VIII.
"Yo no me di mala vida. De mi estado emocional dependían muchos factores, de que mis hijas estuvieran bien. Entonces yo trataba de estar lo más tranquila posible, colaboraba con las

enfermeras, les ayudaba a hacer cosas porque no tenía nada más qué hacer *(durante la hospitalización)*". (C2)
"Como tenía el plan premium, tenía acceso directo a los gineco-obstetras. Digamos que no tuve que dar tanta vuelta como sé que a otras mujeres les pasa". "Que puedas tener un teléfono, o un pediatra, o un gineco-obstetra especifico… algo mucho más cercano, que tenga que pedir una cita y que tenga que hacer desplazamiento. Es que a veces tanta tramitología es un poco compleja para la situación de un estado de embarazo". "Todo el tema de la lactancia que tampoco fue fácil… los consejos de las enfermeras en ese sentido porque a mí al principio no me salía la leche, eso realmente a mí me ayudó mucho… de que me enseñaran". "Dejaron que mi esposo estuviera en cada momento del proceso, inclusive, que lo dejaran entrar al parto sin mayores problemas". (C3)
"Fue una inseminación, o sea que es buscado, y lo consigues, pues claro es la mayor felicidad. Y entonces si tienes un embarazo super bueno, que yo no tuve vómitos, o sea yo podía hacer vida normal. Entonces, estás mucho más motivada porque puedes hacer tu vida normal y encima vas a tener un bebé". (S1)
"Hay que hablar y decir lo que uno siente como madre, y que te tomen en cuenta". "La predisposición, a cambiar un poco tu rutina. A la hora de estar embarazada pues hay que hacer una serie de cosas como para la alimentación y tomar la medicina". (S2)
"Yo no estaba tan nerviosa y era más consciente de todo lo que estaba pasando". "Una amiga que trabaja en un hospital me dijo: vente y vamos a hablar con el ginecólogo, a ver qué pasa". "Enseguida me mandaron a un médico. Luego tuve un par de problemas con la peque porque le dieron como unos ataques epilépticos y enseguida la médica me dijo pues vamos al psicólogo y me han estado tratando súper bien". (S3)

Transcript IX.
"Si tu trabajas bajo estrés… al final lo pagas con quien menos debe en situaciones muy delicadas, porque el parto es una situación que tienes que coger con pinza porque cada caso es una historia diferente, y no puedes tratar a la gente mal poque tu tengas un mal día". "Es que ellas te cosen eso *(vagina)*, y ya no te lo revisan. Que la ginecóloga antes de dar el alta me miró: esto lo tienes perfecto; ¿cómo perfecto?, luego yo estuve preocupada". (S1)
"Deben tener un poquito más de cuidado para tratar… como que de ponerse en el lugar de la mujer que recién ha parido y enseñarle". (S2)
"Venía el matrón y era: a mí me gustan los partos rápidos y sin dolor… y yo: a mí también, pero ya está, ya no me decía nada más. Y yo sólo me quedaba con el rápido y sin dolor". (S3)

Transcript X.
"Cada paciente debe tener una atención un poco más personalizada porque yo entiendo que cada caso es diferente. A lo mejor lo que yo necesito no lo necesita la persona que tiene al frente. No pueden tratar a todos por igual". (S1)
"Me dijeron: ¿quieres verlo? Yo: sí. Me pusieron un espejo para poder verlo… fue fenomenal. Y de hecho me dijeron: ahora cuando vaya a salir, pon las manos que la vas a coger tú. Y yo: ¿pero en serio? Y conforme salió la cogí con las manos y yo me la puse en el pecho. Entonces es como que tengo todo el recuerdo superbonito". (S3)

Transcript XI.

“les pagan muy tarde… o son horarios muy largos y están cansados. Si a ellos les vulneran sus derechos, pues ellos no van a llegar con la mejor actitud del mundo a atender”. (C2)

“Que les capaciten un poco mejor en la atención… un módulo de humanidad, de cómo tratar a la persona desde lo psicológico, desde lo sentimental, más allá del procedimiento”. (C3)

“Yo esperaba también que los mismos especialistas te fueran haciendo un seguimiento hasta el final, pero no es verdad”. (S1)

“Sí que es verdad que meten un montón de presión de: tienes que tener a tantos pacientes al día…que metan a más médicos, al final no pueden hacer milagros ni te pueden atender en 1 minuto, es imposible”. (S3)
